# Adapting a Physical Earthquake-Aftershock Model to Simulate the Spread of COVID-19

**DOI:** 10.3390/ijerph192416527

**Published:** 2022-12-09

**Authors:** Thanushika Gunatilake, Stephen A. Miller

**Affiliations:** 1 Center for Hydrogeology and Geothermics (CHYN), University of Neuchâtel, 2000 Neuchâtel, Switzerland; 2 Swiss Seismological Service (SED), ETH Zürich, 8092 Zürich, Switzerland

**Keywords:** COVID-19, modelling pandemic, earthquake aftershock model, non-linear diffusion

## Abstract

There exists a need for a simple, deterministic, scalable, and accurate model that captures the dominant physics of pandemic propagation. We propose such a model by adapting a physical earthquake/aftershock model to COVID-19. The aftershock model revealed the physical basis for the statistical Epidemic Type Aftershock Sequence (ETAS) model as a highly non-linear diffusion process, thus permitting a grafting of the underlying physical equations into a formulation for calculating infection pressure propagation in a pandemic-type model. Our model shows that the COVID-19 pandemic propagates through an analogous porous media with hydraulic properties approximating beach sand and water. Model results show good correlations with reported cumulative infections for all cases studied. In alphabetical order, these include Austria, Belgium, Brazil, France, Germany, Italy, New Zealand, Melbourne (AU), Spain, Sweden, Switzerland, the UK, and the USA. Importantly, the model is predominantly controlled by one parameter (α), which modulates the societal recovery from the spread of the virus. The obtained recovery times for the different pandemic waves vary considerably from country to country and are reflected in the temporal evolution of registered infections. These results provide an intuition-based approach to designing and implementing mitigation measures, with predictive capabilities for various mitigation scenarios.

## 1. Introduction

The global COVID-19 pandemic demonstrates that modelling the infection propagation is essential for managing and mitigating its spread and containment [1,2,3]. Models describing infections during pandemics can be roughly separated into three categories; (1) the widely-used SEIR (Susceptible–Exposed–Infected–Removed) model [4,5,6], and many of its variations [7,8]; (2) ABM models [9,10,11,12,13]; and (3) models based on reaction-diffusion processes [14,15,16]. The SEIR model couples sets of ordinary differential equations constrained by numerous variables, including the important (but difficult to constrain [17]) infection rate (R), to produce predictive outcomes. ABM models track up to 6.5 billion numerical people interacting with and infecting other numerical people based on (uncertain) rules of human behavior. Other approaches include concepts of self-organized criticality (SOC) [18], or Monte Carlo simulations [19]. Each of these modelling approaches contain multiple and sometimes intractable variables [6,17], resulting in large uncertainties in outcomes, thus restricting their utility in guiding local, national, and international governmental decisions for managing and controlling pandemics.

We propose a different perspective for viewing pandemic propagation and present a physical and numerical model that we consider simple because it contains fewer parameters. We view this simplicity as a benefit because uncertainty rapidly grows with parameter space. Therefore, we adapt a simple model [20] of non-linear fluid pressure diffusion through a porous media to the COVID-19 problem, which was originally developed to simulate aftershock sequences of earthquakes with superior fits to data than the well-known empirical Omori–Utsu Law of aftershocks [21,22]. Since pandemics are large-scale epidemics, a physical aftershock model can be viewed as an epidemic/pandemic model. The basic assumption of the Epidemic Type Aftershock Sequence (ETAS) model is that aftershocks can generate their own aftershock sequences [23,24,25]. In this work, we do not use the formulation for the ETAS model, which is a point-process based on established empirical laws. Nevertheless, the ETAS reference is still relevant using the following logic: (1) the point-process ETAS model has been used for decades to match the rate of aftershocks following large earthquakes. It uses well-established empirical laws to match data; (2) a physical model [20,26] reproduces aftershock rates at least as well as the ETAS model; therefore, (3) the physical model is analogous to ETAS even though they are founded on entirely different formulations. We do not assume ETAS in our model, but ETAS-type behavior emerges from the underlying physics. We have, thus, adapted our physical aftershock model to a Pandemic Type Aftershock Sequence (PTAS) model because infected humans can infect other humans, just as aftershocks can generate their own daughter aftershocks.

In addition, the physical earthquake/aftershock model showed that aftershock decay rates are controlled by the tectonic ability to heal co-seismic and post-seismically generated permeability networks. In the pandemic analog, the decay of infection rates are determined by the societal ability to suppress infection networks through mitigation measures.

## 2. Physical Model

Diffusion of fluid pressure in a porous medium is governed by [20,26]:

(1) $$\frac{dP}{dt}=\frac{1}{\phi \beta }▽\cdot \left[\frac{k}{\eta }▽P\right]+\frac{Q}{\phi \beta }$$

where *P* is fluid pressure [Pa] above hydrostatic, *t* is time [s], ϕ is porosity [-], β is compressibility [Pa${}^{-1}$], *k* is the permeability [m${}^{2}$], η is fluid viscosity [Pa s], and *Q* is a source term [s${}^{-1}$].

In the pandemic analogy, we apply an infection source rate ($S_{i}$), and calculate the time evolution of infection pressure ($P_{i}$) as it diffuses through societies. Thus, infection pressure is an abstract quantity that reflects the spread of the virus and can be written as:

(2) $$\frac{dP_{i}}{dt}=\frac{1}{\phi \beta }▽\cdot \left[\frac{k_{i}}{\eta }▽P_{i}\right]+\frac{S_{i}}{\phi \beta }$$

where $P_{i}$ is infection pressure [Pa], *t* is time [s], ϕ is a measure of the population distribution [-], β is compressibility [Pa${}^{-1}$] interpreted as societal compliance, $k_{i}$ is the infection permeability [m${}^{2}$] reflecting the resistance to infection pressure gradients, η [Pa s] is the viscous term describing the ease of flow (e.g., internal friction), and $S_{i}$ [s${}^{-1}$] is the infection source rate. We purposely preserve, for clarity, the physical units of the porous media analogy.

The diffusivity κ reflects the rate of infection pressure propagation throughout each country:

(3) $$\kappa =\frac{k_{i}}{\eta \phi \beta }$$

while permeability is further defined as:

(4) $$k_{i}=k_{0}e^{\pm \alpha_{j}t_{j}}$$

where $k_{0}$ [m${}^{2}$] is the initial resistance to infection diffusion. A reduction in $k_{0}$ over a timescale $\alpha_{j}$ [s${}^{-1}$] reflects the increased resistance to flow in response to mitigation measures. Note that diffusivity, which is composed of the dynamics of permeability, viscosity, compressibility, and porosity, accounts for the reduction in usage of public transport, enforced shutdowns, mask requirements, and social distancing controlled by $\alpha_{j}$ over the timescale of $t_{j}$.

The source term $S_{i}$ is defined in similar way:

(5) $$S_{i}=Q_{0}e^{\pm \zeta_{j}t_{j}}$$

where $Q_{0}$ [s${}^{-1}$] is introduced throughout the domain. Initially, the source term is concentrated at airports and major transportation hubs because that is where the virus entered. The source term takes a positive value when the virus enters the system and a negative sign in response to mitigation measures. $\zeta_{j}$ [s${}^{-1}$] as used in Equation (5) reflects the response of infection pressure sources based on mitigation measures.

We distinguish between $\alpha_{j}$ (Equation (4)) and $\zeta_{j}$ (Equation (5)) to allow for their exploration independently in future studies, but in this work we assume $\alpha_{j}=\zeta_{j}$ to limit the number of free parameters used to fit the data. Equation (2) is the mathematical formulation for calculating infection pressure propagation, so *P* in Equation (2) reflects the intensity of the virus in the area. When the pressure propagates through the model and reaches an arbitrary (but constant) critical state, which is defined by a threshold, numerical infections are triggered, accumulated, and counted. The threshold is derived from the physical earthquake model. In physical mechanics, the changes in stresses in each direction are considered to calculate the point of rupture at each time step, which results in a numerical earthquake. In those studies, we found that the average value for generating an earthquake is about 1 MPa. In a pandemic analogy, we do not have tectonic stress fluctuations and assume 1 MPa as the initial threshold. Once the threshold is reached, i.e., the viral load is higher at a certain point, that grid point is considered infected. From a physical earthquake perspective, a numerical earthquake can generate its own earthquakes, known as daughter aftershocks. The chain reaction of infections is included in our model because the permeability of the nodal grid point where the numerical infection was triggered is recalculated at each time step. As a result, infection pressure continues to propagate and causes further infections. The growth and decay of infections are subsequently controlled by diffusivity, with pressure gradients diffusing along high diffusivity pathways (e.g., high-speed trains, high population density, etc.). We compare normalized cumulative numerical infections with the number of reported cases in each country. The cumulative number of infections in a country can be best modeled by considering the heterogeneity of the entire system. For example, the transport infrastructure and population density are included in the diffusivity, which controls the rate of infection pressure propagation.

All parameters in all simulations were defined a priori and remained unchanged throughout the simulation, with the exception of $\alpha_{j}$. We intentionally restricted our parameter choices to maintain our analogy with the porous media counterpart, which constrains permeability, viscosity, and compressibility to limited ranges. The terms $\alpha_{j}$ are included in both the permeability and source term, and control the permeability changes at the onset of infection, their recovery, and the amount of source (viral load) over the time scale. They are our fitting parameters and are constrained by the data for each observed infection wave. $\alpha_{j}$ in Equations (4) and (5) modulates the system compliance ϕβ in both the diffusion and source terms, and dominates the model behavior. The concavity observed in the data constrains the sign of $\alpha_{j}$. Conceptually we might decompose β into political compliance $\beta_{p}$ and economic compliance to $\beta_{e}$ because a country’s economic health might also affect a country’s response. Such a decomposition is analogous to a separation between fluid compressibility and pore space compressibility in the earthquake model. In this work, β is unconstrained, so we assume a lumped compressibility with a constant value of ($\beta =10^{-8}$ Pa${}^{-1}$) for all countries. We use the porosity in the model as a measure of population density. We define porosity as $\phi =0.5-\left(\frac{f_{s}}{f_{c}}\right)$, where $f_{s}$ is the population of a state and $f_{c}$ is the population of the entire country. The 0.5 term is constrained by demographics and limits the range of ϕ to reasonable values of the model’s geological analog (0.2<ϕ<0.49). For each simulation, we defined parameters ϕ, *k*, η, and *S*, for each state within that country. Parameter space was extensively investigated, and we arrived at the parameters that best matched the data. The initial permeability $k_{0}$ takes on values of either $10^{-12}$ m${}^{2}$ or $10^{-13}$ m${}^{2}$, with the former applied to high population density and their corresponding transportation networks, and the latter for sparsely populated regions. The viscous term (η) takes on values of $10^{-3}$ or $10^{-2}$ Pa s with the lower value reflecting the degree of public transport use. As an example, more than 20% of the population in New York and California used public transport regularly prior to the pandemic and are, thus, assigned $\eta =10^{-3}$ Pa s. Conversely, the populations of Utah and Oklahoma rarely used public transportation prior to the pandemic and are, thus, assigned an order of magnitude higher viscosity using $\eta =10^{-2}$ Pa s. In addition, New York and California also had a higher influx of visitors prior to the pandemic, so we assume a higher initial permeability $k_{0}=10^{-12}$ m${}^{2}$ compared to $k_{0}=10^{-13}$ m${}^{2}$ for Utah and Oklahoma. Finally, *Q* is $10^{-8}$ s${}^{-1}$ at points of entry and $10^{-9}$ s${}^{-1}$ throughout the remainder of the domain and is mostly the same on average for all studied cases (Supplementary Figure S3). These values of all parameters were chosen to mirror (to some degree) their geological analog, and interestingly, the initial values best-suited for this model of infection pressure propagation have the hydraulic properties of water and beach sand as their porous media counterpart. Supplementary Table S1 lists the source of our input for population demographics and the viscous term η, which we equate to the use of public transportation and the probability of human interaction.

**Figure 1:**
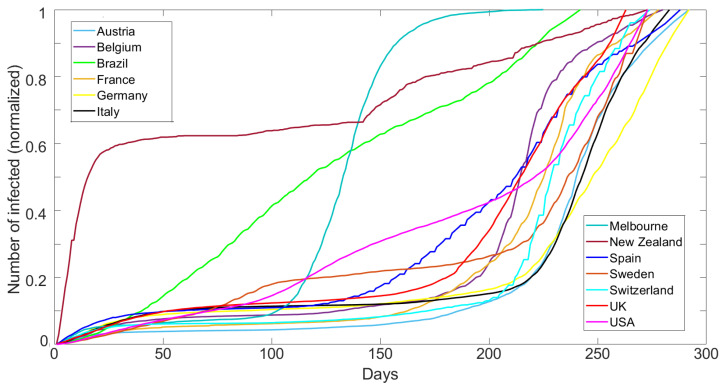
Compilation of reported cumulative infections for all cases studied and identified in the legend. The time range was 15 March 2020 to 4 January 2021 for all countries (Source: ECDC and Australian Government).

Figure 1 shows the cumulative number of reported cases normalized by the maximum reported cases for each dataset over 300 days (15 March 2020 to 4 January 2021) for Austria, Belgium, Brazil, France, Germany, Italy, Melbourne (AU), New Zealand, Spain, Sweden, Switzerland, UK, and the USA. The data are published by the European Centre for Disease Prevention and Control (ECDC) [27], which monitors the COVID-19 pandemic. The Melbourne data were obtained from the Australian government of health and human services [28]. The data show a very broad range of behavior, which we show below to be modeled only by varying $\alpha_{j}$.

The numerical model used in this study is the same as described in reference [20], with the addition now of a source term. The source term was also recently incorporated into the aftershock model and applied to the Central Apennines [26]. It is useful to describe in detail the model setup for Switzerland as one particular case, which is then applied to all other simulations. The Swiss Confederation consists of 26 member states, and each state is implemented with its geographical boundaries. The often-used transportation network primarily links the major cities of Geneva, Lausanne, Basel, Zurich, and Lugano, and is modeled as a highly permeable, low-viscosity channel within the model. Additionally, significant airport hubs as points of entry, ranked by passengers per year, are included as groups of nodal points with high infection source rates. These statistics (for 2020) are provided by the Swiss federal office, which also provides population density and percentage of public transport use per federal state. At time zero, infection sources at points of entry develop infection pressure that then diffuses over time along infection pressure gradients and through highly permeable channels, triggering model infections along the way. The model setup of the 11 other countries and Melbourne are defined using this same procedure. This initialization of model geometry establishes domains of population density, transportation networks, etc., that controls the initial spread of the virus, and we assign the corresponding parameters (ϕ, β, *k*, η, and *S*). The design of each model is based on the infrastructure, considering all states as a subunit of each country. Infections can propagate throughout the model, and the no flow boundary conditions are applied along all boundaries (e.g., border closures). We numerically solve Equation (2) using implicit finite differences on a regular grid of 300 × 300 nodal points, and calculate infection pressure diffusion in the numerical domain associated with each country. In the aftershock model [20], the triggering threshold is defined by the Mohr–Coulomb failure condition, but no such similar constraint exists on infection triggering. Therefore, we arbitrarily set the threshold to 1 MPa for all simulations. When the threshold is reached, time ($t_{i}$) in Equations (4) and (5) initiates at that numerical grid point, which influences subsequent solution of Equation (2). To allow multiple infections at the same nodal point, we double the threshold necessary to trigger each subsequent infection. Heterogeneity is inherent in the model through the characteristics of each country.

## 3. Results

**Figure 2:**
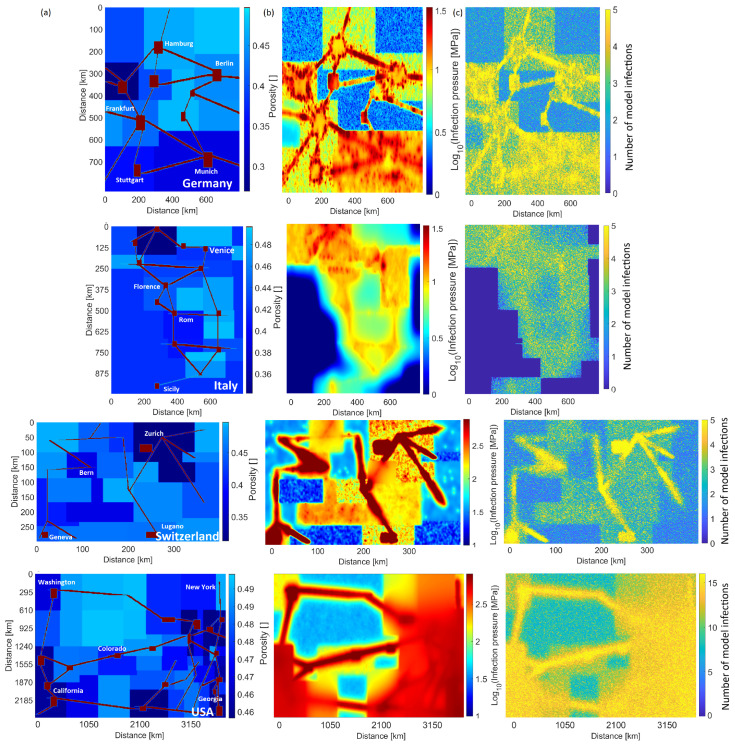
(**a**) Model setup for Germany, Italy, Switzerland, and the USA showing the source locations (red boxes) signifying airports and intercity rail lines (red lines), and the various shades of blue scale with population density reflecting the “porosity” distribution and delineate federal states. (**b**) Calculated infection pressure at the end of the 300 day simulation. Note change in scale bar for each country because of the very large variations in infection pressure. (**c**) The number of repeated infections calculated in the model highlights the regions most affected and shows how elevated infection pressures (**b**) continue to generate model infections. Similar plots for all other cases studied are found in Supplementary Information.

From this input, the initial conditions at the start of each simulation (Figure 2a) are heterogeneous and approximate at a large scale the overall societal setup. We use a timestep of one day, which results in a total simulation time of about 2 min on a typical laptop running a MATLAB script (We also tested timesteps of 430 s, with no change in results). Our comparisons with data extend to 300 days, which covers the onset of the pandemic in each country until both virus mutations and the introduction of vaccines modify the datasets in yet unknown ways.

Figure 2b,c show typical model results for four different countries (see Supplementary Figures S1 and S2 for the remaining cases). The calculated infection pressure concentrates in large urban areas (Figure 2b), reflecting high population density and ease of flow (e.g., viscosity) but also shows pervasive elevated pressures throughout each country. This figure visualises the dramatic differences in infection pressure (and thus modeled infections) for the different countries. Unsurprisingly, the calculated number of cumulative infections also correlates with population concentration and ease of flow (Figure 2c).

**Figure 3:**
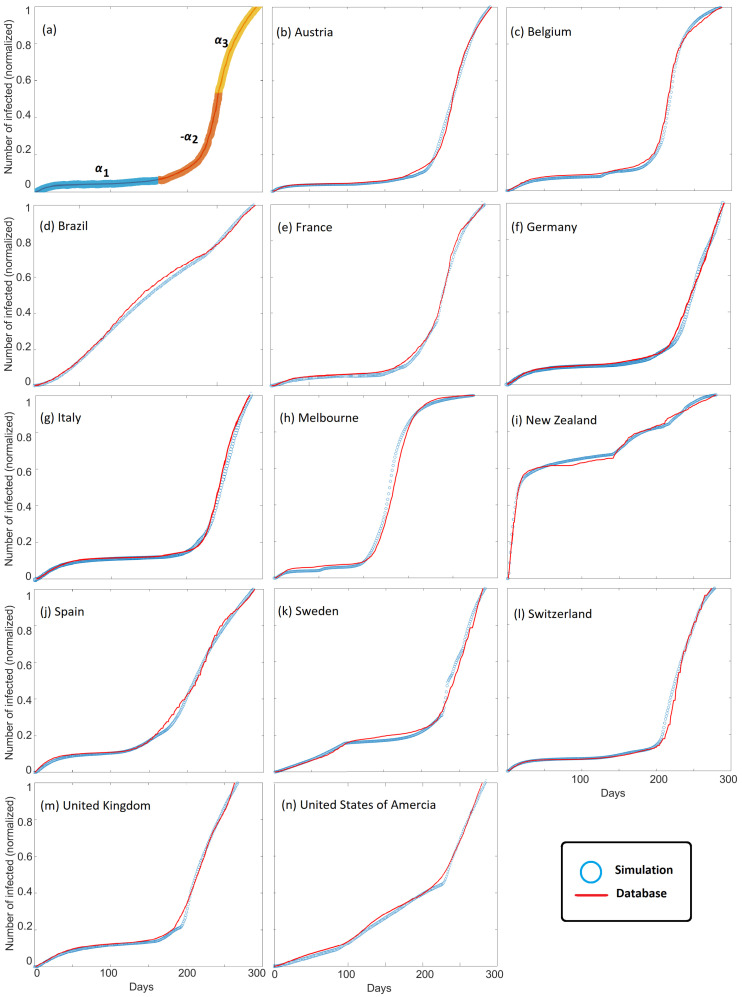
Comparison of all data (red) in Figure 1 superposed with the corresponding model results (blue). The data constrains ±α=±ζ and how this is achieved is demonstrated in (**a**). The colors in (**a**) represent the duration of each phase. Excellent agreement in all cases is observed.

Figure 3 shows the observed cumulative infections (i.e., Figure 1) superposed with modeled cumulative infections for all countries studied. Good agreement between model and observations is found for all countries, and model scaling is demonstrated by comparing results with observations at the local scale of Melbourne, Australia. We chose Melbourne, Australia, because they underwent two severe lockdowns. The model successfully matches cumulative infection observations for large countries, small countries, and states. This agreement is observed despite the vast differences in governmental and societal response, and importantly, good agreement is achieved by modeling the parameter α, with different values for α chosen to fit the dynamics of COVID-19 propagation (Figure 3a). The parameter α dominates the model behavior because it modulates the system compliance ϕβ that appears in both the diffusion and source terms in Equation (2).

**Figure 4:**
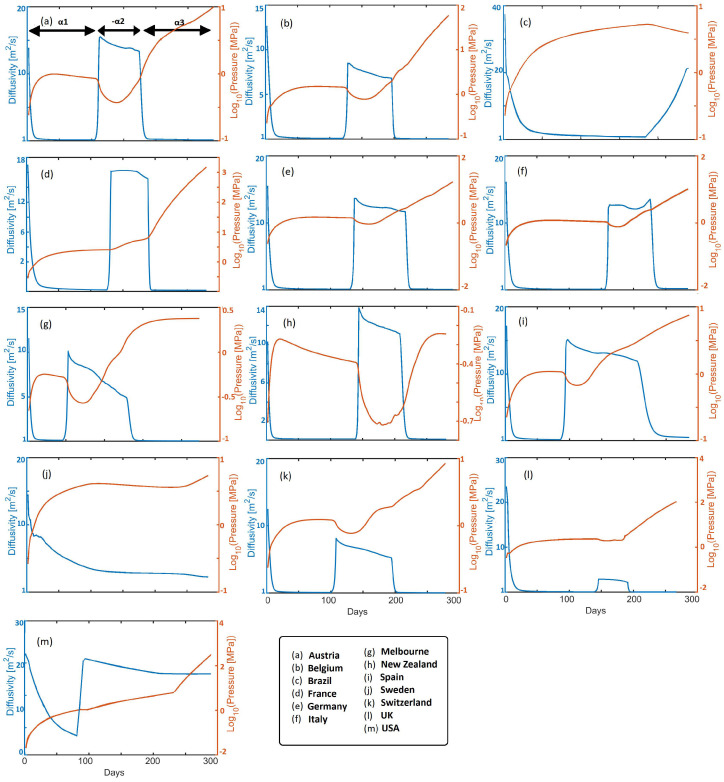
Modeled diffusivity (blue) and pressure time histories (orange) for each individual country (and Melbourne) showing the hydro-dynamical response to mitigation measures. For reference, the hydrogeological properties of beach sand and water is roughly 15 m${}^{2}$ s${}^{-1}$.

Figure 4 shows time histories of calculated infection pressure $P_{i}$ and diffusivity κ, for all cases studied. The diffusivity reflects the rate of infection pressure propagation throughout each country (3). For intuitive reference the diffusivity of water and beach sand is roughly 15 [m${}^{2}$ s${}^{-1}$], so from a physics perspective, the virus propagates very quickly. The initial rapid drops in diffusivity reflect pro-active societal response to government measures in all presented countries. Reduced diffusivity consequently results in rising infection pressure and pressure gradients, which remain in the system, to then subsequently diffuse upon relaxation of mitigation measures. Looking at an exemplary EU state (e.g., Austria), the diffusivity initially decreases to a value of approximately 2 [m${}^{2}$ s${}^{-1}$] in response to mitigation efforts. Meanwhile, pressure increases over this timescale constrained by ζ (where ζ=α in this study) in Equation (5), and which subsequently diffuses, resulting in a mild pressure decrease. A sudden rise in κ correlates with imposition of $\alpha_{2}$, and, thus, the consequent pressure drop, followed by a mild apparent reduction in κ. Imposition of $\alpha_{3}$ correlates with mitigation efforts that again reduce κ and increase $P_{i}$. This results in the onset of the 2nd wave, which we model by imposing $-\alpha_{2}$ that dramatically increases diffusivity and the consequent reduction in $P_{i}$. Finally, $\alpha_{3}$ reflects additional mitigation measures, and subsequent waves can be modeled with additional values for α. Note that infection pressure can continue to increase during the healing process because of the exponential time-dependence of the source term.

**Figure 5:**
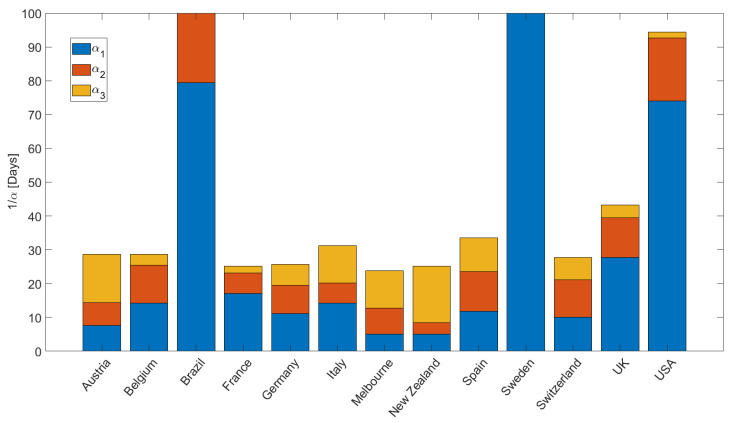
Summary of α values used in the simulations, showing a wide range of recovery times for different countries. $\alpha_{1}$ (blue) represents the recovery time of the first wave, $\alpha_{2}$ (orange) represents the second wave, and $\alpha_{3}$ (yellow) represents the recovery of the on-going third wave.

Figure 5 quantifies the values for α, plotted for intuitive convenience as 1/α [days], used in the simulations to fit the data (e.g., Figure 3). We note a few important points. First, Brazil, Sweden, and USA reveal the longest recovery times $1/\alpha_{1}$, indicating that people are exposed to the virus longer. The modeling results show that Italy, Spain, and the United Kingdom have longer recovery times (slightly more than one month) than the other EU countries (and Switzerland), where recovery times are approximately less than two weeks. The fastest recovery times were observed for Austria, New Zealand, and Melbourne. The acceleration in infections at the onset of the 2nd wave is quantified by $-1/\alpha_{2}$, and shows rapid acceleration in Belgium, USA, Spain, Switzerland, and the UK. This acceleration during the second wave is explained in the model as the onset of diffusion (instigated by relaxation of mitigation measures) of latent infection pressure gradients stored in the system. Finally, $\alpha_{3}$ reflects the ongoing situation, and may change depending on governmental measures and societal response. Note that the concavity for New Zealand at the onset of the second wave (Figure 3i) required $+\alpha_{2}$, and that Sweden and Brazil had essentially no recovery from the first wave, so $\alpha_{2}$ and $\alpha_{3}$ are essentially zero.

## 4. Discussion and Conclusions

We presented a simple model for the propagation of infection pressure through societies and compared model results with global COVID-19 data. We find good agreement for all cases studied, and importantly, fits to the data are achieved by varying parameter α, where α is included in the diffusion and source terms in Equation (2) and quantifies the response to mitigation efforts. The purpose of this work is to present a new formulation of infection propagation using a deterministic model, and which matches a very wide range of data using only a few (and intuitive) parameters. The result that only slight variations in model parameters can reproduce all observations of infection rates across the globe strongly indicates that this model captures the essence of the physics of pandemics. This physical deterministic model reproduces the cumulative number of COVID-19 cases in a dozen countries including an island (New Zealand), and a confined city (Melbourne, AU) to demonstrate that this model scales. We have shown it to be successful in matching data for large countries, small countries, and countries with wide ranging social–political–economic conditions. The model itself is descriptive in that it explains the distribution of the virus based on different population densities, different numbers of transport options, and different patterns of public transport use.

Finally, the model suggests that future strategies should be explored for reducing the latent infection pressure remaining in societies after successful mitigation measures. Therefore, compressibility, which in physical terms can be divided into fluid and rock compressibility, can be separated into political and economic compliance. The model would be capable of predicting the perspective evolution depending on the evolution of these two compliances. Based on this knowledge and incorporating parameters, such as political restrictions (e.g., lockdowns, masks, etc.) and vaccination probabilities, into the physical notion of compressibility, we can predict the temporal and spatial evolution of the virus in future studies. Furthermore, the influence of mutations can be addressed in future modeling, which our findings suggest may be further advanced by invasion percolation approaches. Once the model has been calibrated against observations (at any time), parameters can be varied to predict alternative outcomes. This is the utility of our model because we can simulate different mitigation scenarios to optimize mitigation measures and bleed-off of infection pressure without overwhelming society.

For a general understanding of our findings, imagine the following: you are sitting on the beach with a hollow steel pipe, sealed at the bottom but with holes along part of its length. The part of the pipe with holes is driven into the sand. Water is then poured into the hollow pipe. The fluid quickly diffuses into the sand (infection propagation), and at rates that depend on the height of the water column in the steel pipe. At some distance from the pipe, a metal barrier surrounding the pipe is inserted into the sand (e.g., masks, lockdown, etc.), and the fluid flow (infection propagation) is curtailed. However, the pressure to drive the system is still in the pipe, so when lockdowns or mask restrictions are eased, fluid pressure (infection) quickly propagates (2nd wave), until mitigation measures are re-applied. Now, imagine further, a number of pipes inserted in the sand (e.g., airports) scattered across regions. Each system acts independently but interacts dynamically with all other pipes inserted into the system. In our model, the initial pipes are the airports and α controls the barriers. With this conceptual understanding, one goal would then be to establish strategies that bleed off the pressure in the pipe (infection pressure in our model) during the lockdown/mask phases so that when the restrictions are eased, there is much less fluid (infection) pressure in the pipe to drive the subsequent wave or waves. Expanding on this, we can envisage an unlimited number of fluid-filled pipes on an infinite beach, each linked in a neural network, and use machine learning or artificial intelligence to predict the optimal scenario for implementing mitigation measures in both space and time across societies.

## Data Availability

The datasets presented in this study are available in the online repositories of the European centre for disease prevention and control ECDC. https://www.ecdc.europa.eu/en/publications-data/download-todays-data-geographic-distribution-covid-19-cases-worldwide (accessed on 4 January 2021).

## Supplementary Materials

The following supporting information can be downloaded at: https://www.mdpi.com/article/10.3390/ijerph192416527/s1.
