# Supplementary material for: Adapting a Physical Earthquake-Aftershock Model to Simulate the Spread of COVID-19
Source: Int J Environ Res Public Health. 2022 Dec 9;19(24):16527. doi: 10.3390/ijerph192416527 (PMC9778620; doi:10.3390/ijerph192416527)
Supplement: Supplementary file 1 [file ijerph-19-16527-s001.zip › ijerph-1998515-SI.pdf]

Supplementary Information for 'Adapting a physical earthquake-aftershock model to simulate the spread of COVID19'

Thanushika Gunatilake and Stephen A. Miller

Country	Website	Link
Austria	Statistik Austria - Die Informationsmanger	https://www.statistik.at/atlas
Belgium	Statbel, the Belgian statistical office	https://statbel.fgov.be/en
Brazil	Instituto Brasileiro de Geografia e Estatistica	https://cidades.ibge.gov.br/brasil
France	Insee - National Institute of Statistics and Economic Studies	https://www.insee.fr/en/accueil
Germany	Statistische Ämter des Bundes und der Länder Gemeinsames Statistikportal	https://www.statistikportal.de/de
Italy	Istat Statistics	https://www.istat.it/en/
Melbourne	Research and statistics - City of Melbourne	https://www.melbourne.vic.gov.au
New Zealand	Stats New Zealand	https://www.stats.govt.nz
Spain	INE- National Statistics Institute. Spain	https://www.ine.es/en/
Sweden	Statistics Sweden	https://www.scb.se/en/
Switzerland	Bundesamt für Statistik-Statistischer Atlas der Schweiz	https://www.atlas.bfs.admin.ch/maps
UK	Research and statistics - GOV.UK	https://www.gov.uk/search/research-and-statistics
USA	United States Census Bureau	https://www.census.gov

Table S1. Sources used in this study.

Supplementary Figure S1

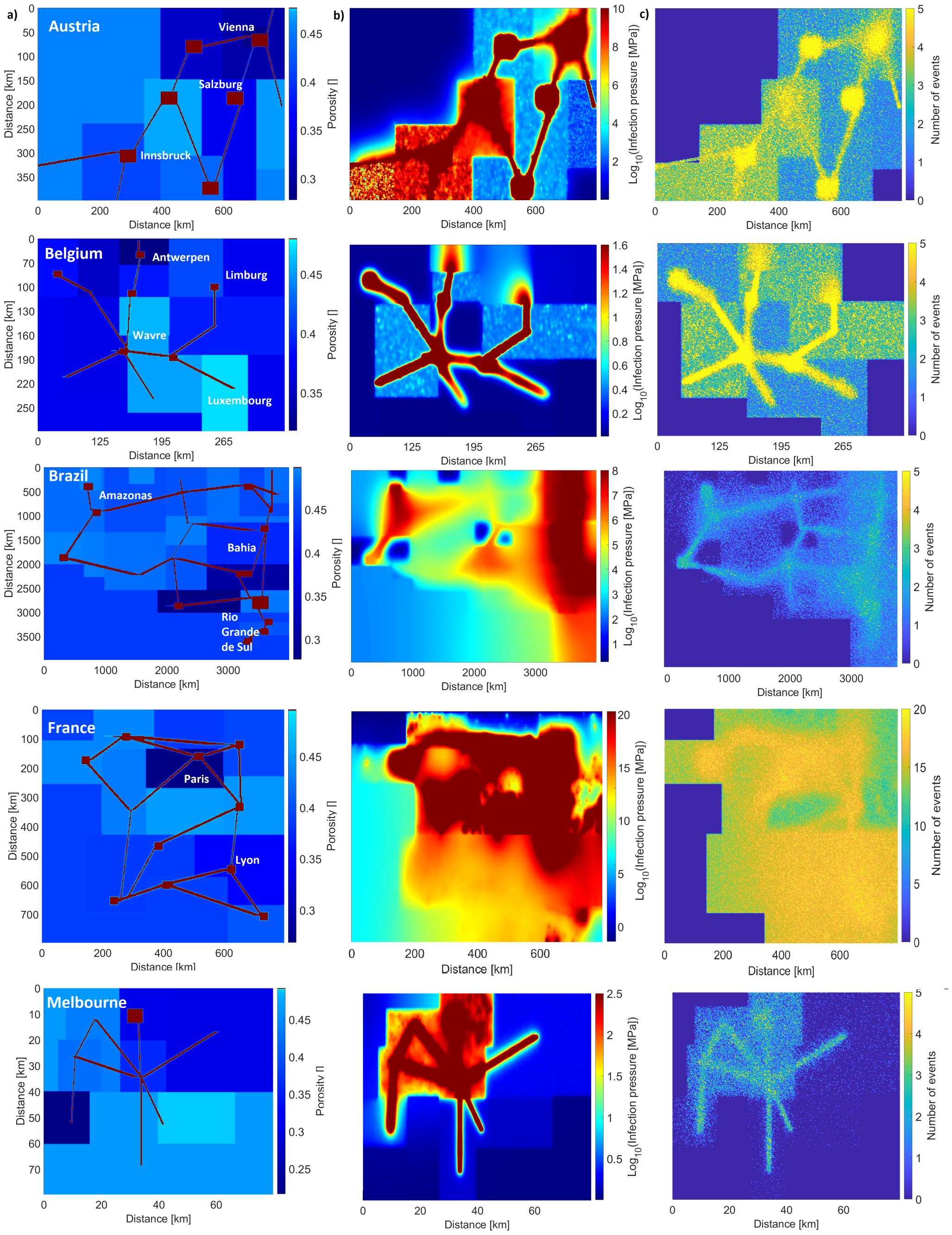

Figure S1: a) Model setup for Austria, Belgium, Brazil, France and Melbourne showing the source locations (red) signifying airports and intercity rail lines, and the various shades of blue scale with population density and delineate federal states. b) Calculated infection pressure at the end of the simulation. Note change in scale bar for each country. c) The number of repeated infections calculated in the model highlights the most affected regions and shows how elevated infection pressures (Figure 1 b) continue to generate model infections.

Supplementary Figure S2

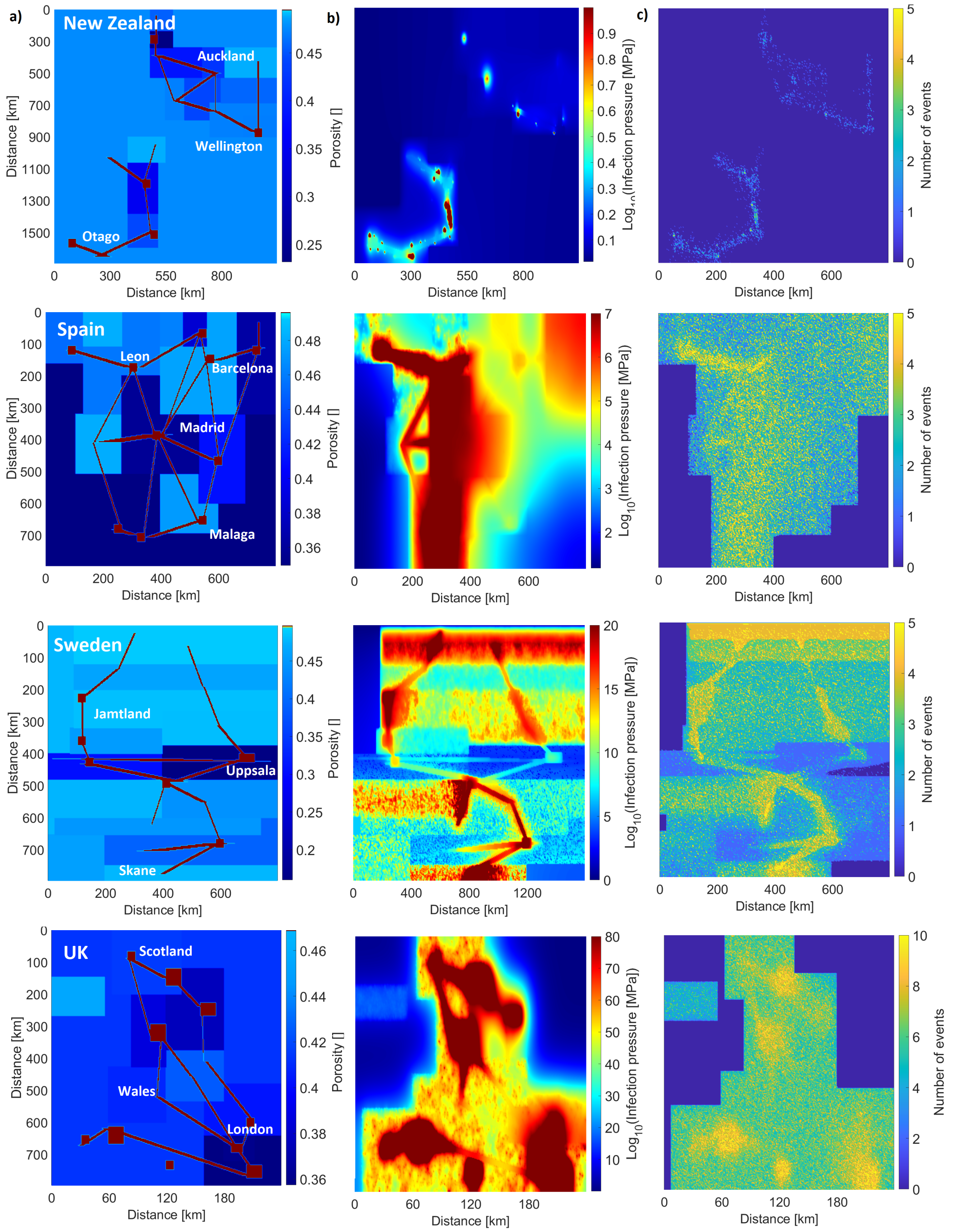

Figure S2: a) Model setup for New Zealand, Spain, Sweden and the UK showing the source locations (red) signifying airports and intercity rail lines, and the various shades of blue scale with population density and delineate federal states. b) Calculated infection pressure at the end of the simulation. Note change in scale bar for each country. c) The number of repeated infections calculated in the model highlights the most affected regions and shows how elevated infection pressures (Figure 2 b) continue to generate model infections.

Supplementary Figure S3

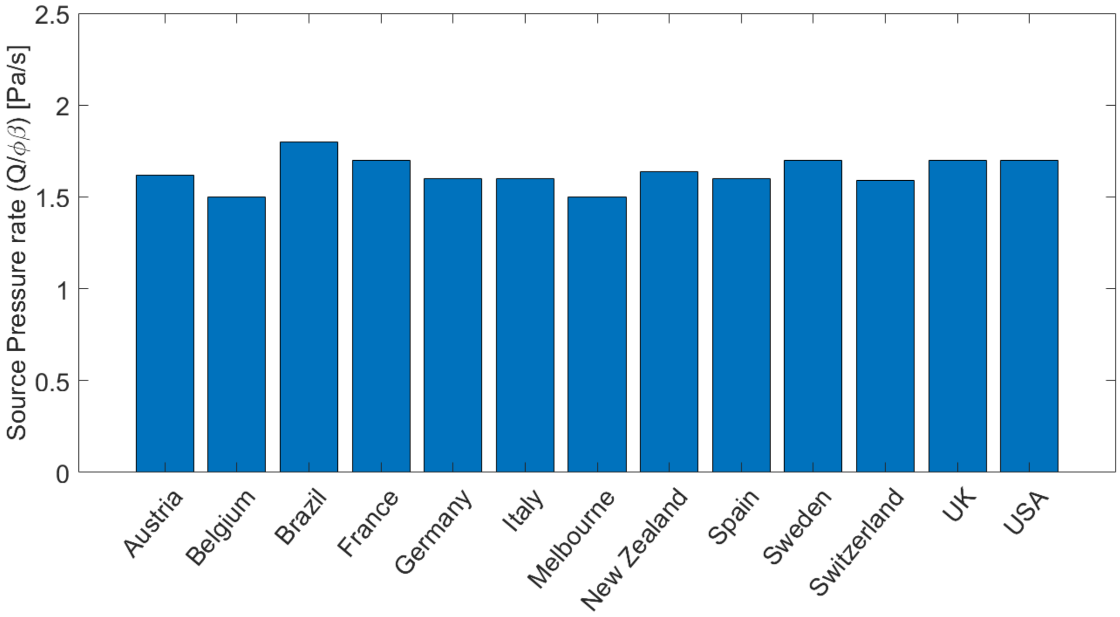

Figure S3: Average initial source pressure rate for each studied case.

Supplementary Figure S4

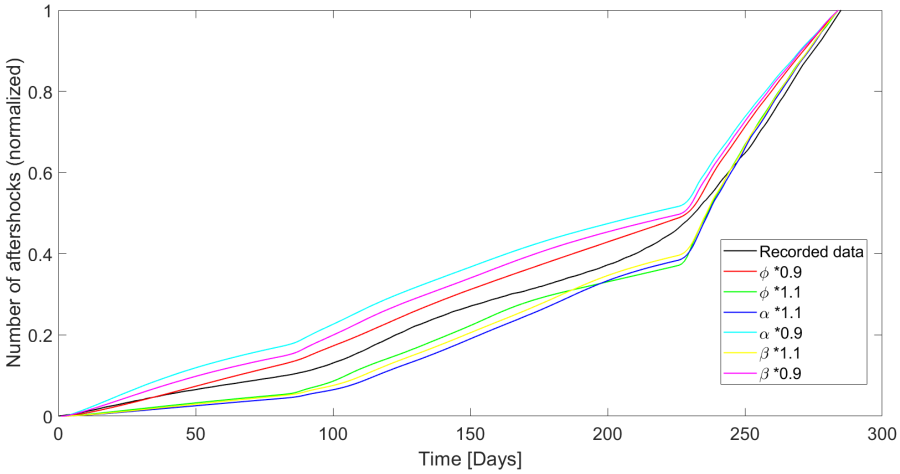

Figure S4: Results with parameter variation for the study case USA. Model results varying α, porosity (ϕ), and compressibility (β) by plus or minus 10% showing poor comparisons with data, demonstrating further tight constraints on model parameters.

Country	Difference
Italy	-0.9%
Germany	-3%
Switzerland	-0.1%
Spain	-3%
UK	-1%
USA	-0.7%
Austria	-0.9%
France	-0.2%
Brazil	-0.5%
Sweden	-0.5%
Belgium	-0.9%
New Zealand	-0.05%
Melbourne	-0.1%

Table S2: Difference between model and observations averaged for the entire record (in percent) for parameter α. We estimate errors by comparing, point for point, the difference between the (normalized) observed cumulative infections, with the (normalized) cumulative model infections. We then take the average of these differences for the entire record.
